# Correction Workers’ Burnout and Outcomes: A Bayesian Network Approach

**DOI:** 10.3390/ijerph16020282

**Published:** 2019-01-20

**Authors:** Jin Lee, Robert Henning, Martin Cherniack

**Affiliations:** 1 Department of Psychological Sciences, Kansas State University, Manhattan, KS 66506, USA; 2 Department of Psychological Sciences, University of Connecticut, Storrs, CT 06269, USA; robert.henning@uconn.edu; 3 Department of Medicine, UConn Health, University of Connecticut, Farmington, CT 06030, USA; cherniack@uchc.edu

**Keywords:** correction workers, stress, exhaustion, psychosocial and behavioral factors, Bayesian Network, Total Worker Health^®^

## Abstract

The present study seeks to demonstrate how Bayesian Network analysis can be used to support Total Worker Health^®^ research on correction workers by (1) revealing the most probable scenario of how psychosocial and behavioral outcome variables in corrections work are interrelated and (2) identifying the key contributing factors of this interdependency relationship within the unique occupational context of corrections work. The data from 353 correction workers from a state department of corrections in the United States were utilized. A Bayesian Network analysis approach was used to probabilistically sort out potential interrelations among various psychosocial and behavioral variables. The identified model revealed that work-related exhaustion may serve as a primary driver of occupational stress and impaired workability, and also that exhaustion limits the ability of correction workers to get regular physical exercise, while their interrelations with depressed mood, a lack of work engagement, and poor work-family balance were also noted. The results suggest the importance of joint consideration of psychosocial and behavioral factors when investigating variables that may impact health and wellbeing of correction workers. Also, they supported the value of adopting the Total Worker Health^®^ framework, a holistic strategy to integrate prevention of work-related injury and illness and the facilitation of worker well-being, when considering integrated health protection and promotion interventions for workers in high-risk occupations.

## 1. Introduction

Correction workers work under psychologically and physically demanding conditions [1,2,3]. They are frequently exposed to unexpected hold-over shift work that may result in circadian disruption, sleep loss, and increased fatigue. Also, correction workers experience constant threat of inmate assaults and riots, which can be emotionally draining due to hyper-alertness and anxiety [4]. Moreover, working conditions in the correctional setting are known to be associated with numerous types of health and performance outcomes, including immediate outcomes, like exhaustion and psychological distress, as well as more distant outcomes, like job dissatisfaction and impaired work ability [5]. According to the U.S. Department of Justice, correction workers, including correctional officers and administrative and support staff, are at higher risk of suicide, substance abuse, and divorce, while their mortality rate is the second highest of any occupation [6]. High turnover rates have also been reported [7].

The negative impact of demanding correctional work on correction workers’ wellbeing can be explained by the classic stress-burnout model [8,9]. Adverse working conditions of correctional work act as stressors causing stress. These stressors lead to psychophysical strain symptoms, such as various forms of psychophysical burnout. Echoing this, empirical studies on correction workers have shown significant relationships between overwork and stress [8,10]. Also, studies have revealed that correction workers’ stress is associated with burnout [8,11] and somatic symptoms [7].

These phenomena can also be understood within theoretical frameworks of the Job Demands-Resources model [12]. Exposure to the physically and psychologically demanding correctional working conditions with a lack of adequate resources is likely to deplete correction workers’ psychological resources for socially adaptive and physically healthy self-regulatory behaviors [13]. Thus, correction workers’ demanding work conditions can lead to exhaustion, which may then negatively influence healthy behaviors as well as socially adaptive behaviors. Additionally, Wright and Cropanzano showed that emotional aspects of exhaustion, which can be manifested in different forms, such as depression and stress, can undermine job performance [14].

In general, the negative impacts of demanding work conditions and subsequent psychophysical strain can be both extensive and derivative. Recent studies have shown various complications of stress. For example, stress can contribute to increased risk of cardiovascular diseases [15], while burnout, as an outcome of extended exposure to stress, can be associated with impairment of psychological resources, such as emotional intelligence and self-efficacy, as well as loss of social support [16]. Moreover, it was shown that burnout can negatively affect well-being at work, resources for coping, ability to work, and engagement [17]. Considering these findings, it can be inferred that specific adverse effects on workers’ health in general (e.g., illnesses, psychological problems, obesity, etc.) are further compounded by compromised family health (e.g., marriage dysfunction, problematic children’s behavior) and employer dissatisfaction (e.g., due to declined performance, increased health care costs). In corrections, work-related stress can lead to increased absentee rates, internal conflict, and suboptimal employee performance [18], all which may have reciprocal adverse influences on the work environment.

Our study attempts to elaborate the Job Demand-Resource model, which primarily focuses on stressors’ impact on stress outcomes (strains), by examining the interrelations among the potential stress outcomes that are typical for correction workers. Our view is that the various stress outcomes do not simply occur in parallel, but instead are able to have synergistic effects when they occur in combination, in line with the view that stressor-strain relationships can be reciprocal [19,20]. Chronic exposure to the occupational context of correctional facilities, with its high demand that also lacks control or support, is likely to lead to various psychosocial and behavioral stress outcomes. In fact, Tennant found that enduring structural occupational stress can contribute to psychological disorders, like depression [21]. When confronted with different types of strain symptoms, one’s resources, which are always limited in the case of correction workers, need to be used to cope with the extant symptoms first, whilst one’s resources may not be adequately restored in a timely manner in order to respond to the emergence of additional strain symptoms. Subsequently, one’s strain symptoms are likely to be exacerbated as more problems arise and as resources become more and more depleted.

The process of resource depletion is particularly concerning in situations in which strain symptoms are not simply end products but operate as derivative or secondary demands (or stressors). As various strain symptoms negatively affect a person in different ways when they are either within or outside the workplace, this suggests a person needs resources, including increased control and support, both within and outside the workplace to handle multiple strain symptoms. This inference aligns with the perspectives of Spillover Theory [22], as well as Conservation of Resources Theory [23]. Furthermore, when there is a lack of adequate resources, the stress-burnout process would accelerate because of the synergistic impact of multiple strain symptoms on resource depletion.

Our study explores the possible derivative or secondary role of strain symptoms as additional stressors. By doing so, the domains of stress intervention are also broadened from focusing exclusively on traditional stressors to also include strain symptoms themselves. This approach is not unlike secondary (i.e., reducing the impact of an injury and disease that has already occurred) or even tertiary (i.e., mitigating the impact of an ongoing injury and illness which has lasting effect) intervention approaches found in medical science and public health [24]. The dire implications of doing nothing to prevent any worsening or exacerbation of the health situation for correction workers was also a motivating factor.

### 1.1. Socio-Technical Systems Framework and Total Worker Health^®^ Paradigm

The present study is based on a socio-technical systems framework which recognizes the interactions between behavior and the design of work system components and their potential to promote workers’ health and safety [25,26]. Specifically, key principles of joint causation within the socio-technical systems framework [26] provide the rationale for examining the interplay among the organizational (i.e., workability, disengagement), social (i.e., work-family balance), psychological (i.e., stress, exhaustion, depression), and behavioral strain symptoms (i.e., limited physical exercises) in relation to the unique occupational context of correctional work. Understanding the interrelatedness of these various attributes can help in efforts to achieve compatibility of the work system’s elements and goals in order to promote better organizational performance as well as employee safety, health, and wellbeing [27,28].

Socio-technical systems approaches emphasize contextual factors whenever relationships are examined among a specific set of variables used to represent the inherent complexity of the workplace. In this regard, the socio-technical systems approach represents a holistic approach that is consistent with the Total Worker Health^®^ framework being advanced by the U.S. National Institute for Occupational Safety and Health (NIOSH) [29]. NIOSH defines Total Worker Health as “policies, programs, and practices that integrate protection from work-related safety and health hazards with promotion of injury and illness prevention efforts to advance worker well-being” [30]. Researchers and practitioners seeking to advance the Total Worker Health agenda are moving beyond conventional health protection and health promotion approaches by undertaking more comprehensive assessments of the health, safety, and wellbeing of workers, and also to design and test more integrated workplace interventions [31].

According to the socio-technical systems framework, how correction workers deal with the unusually demanding occupational conditions in corrections will be a key determinant of how this adversely impacts them. For example, working conditions in corrections may make it harder for correction workers to balance work and personal life. Subsequently, unique patterns of negative spillover effects [32] can be anticipated to impact both the psychosocial and behavioral states of correction workers, such that a disrupted work-family balance may exacerbate one’s depression, stress, healthy eating, regular exercises, engagement to work, and workability. Meanwhile, the Total Worker Health paradigm speaks to the importance of integrated efforts both within (e.g., management commitment, working environment improvement) and outside the workplace (e.g., support from home and community) in order to effectively manage the adverse impact of the stressful work environment of corrections.

### 1.2. Analytic Approach: Bayesian Network Analysis

Bayesian Network analysis examines the relationships among variables based on their joint probability [33]. It utilizes machine learning algorithms that can efficiently cope with the uncertainty and complexity of component interactions within a system as a whole [34]. Bayesian Network analysis offers a network diagram (directed acyclic graph), which consists of nodes and arrows. The nodes are random variables that may consist of observed continuous or categorical quantities, or even latent variables. The arrows (i.e., edges or arcs) indicate probabilistic relationships. If two nodes are connected with an arrow, this suggests that the two nodes are conditionally dependent.

Bayesian Network analysis is efficient in examining the interactions of all system components included in the model, when individual, organizational, and physical factors co-exist and can jointly contribute, and this has the potential to provide valuable insights on the determinants of employee safety and health outcomes. This approach is remarkably suitable to socio-technical systems approaches, which emphasize the importance of examining the interplay among various system components in order to optimize their functioning for a healthy and sustainable workplace. The structural learning algorithm of Bayesian Network analysis is a type of greedy search algorithm [35], and this approach enables investigation of every possible structural association among selected variables to estimate the most probable model that satisfies the model selection criteria of a particular learning algorithm.

Moreover, Bayesian Network analysis is less susceptible to multicollinearity problems [36]. By leveraging the inter-correlations among variables, Bayesian Network analysis provides conditional probability distributions for the dependent relationships of study variables. A complex system can thus be viewed in a modular way by “breaking down the discovery process into the search for the specific components of a complex system, and thus avoiding…multicollinearity” (p. 59) [37].

Bayesian Network analysis has been successfully applied in various settings that require quantitative modeling of complicated relationships among many variables, such as in genetic modeling and disease diagnosis [37]. Also, the Bayesian Network approach has already been used to unveil the mutual relationships among various organizational and psychosocial factors regarding stressors, stress, and strain in workplace. Specifically, more task demands were associated with more stress at work [38], while social support from both supervisors and co-workers was critical in workplace stress prevention [39]. All things considered, Bayesian Network analysis is therefore well suited to investigate complicated interrelationships among multiple psychosocial and behavioral outcomes of stress.

In summary, it is worth noting that there has been no scientific study which integrates the conceptual framework of Total Worker Health and the machine learning analytic framework as a means to promote workplace health and well-being. No publication was found under the keywords search of “Total Worker Health” and “machine learning” in PubMed and PsycINFO. To address this gap, the present study adopted the Total Worker Health framework and Bayesian Network analysis approach (1) to reveal the most probable ways that psychosocial and behavioral outcome variables in corrections work are interrelated, and (2) to identify the key contributing factors of this interdependency relationship within the unique occupational context of correctional work.

## 2. Method

### 2.1. Participants

Paper-and-pencil surveys were distributed at the correctional facilities with high (level 4) to highest (level 5) security. The data from 353 employees at the Connecticut Department of Correction was obtained from a study conducted by the Center for the Promotion of Health in New England Workplace (CPH-NEW), a NIOSH center of excellence for Total Worker Health. In return for completing the survey, monetary incentives were offered. Mean age of the sample was 42.84 (SD = 9.72) and 74.6% were males. Average job tenure was 11.96 yrs (SD = 6.46). In terms of ethnicity, 71.5% were white, 15.8% were black, and the remaining 12.7% included Hispanic, Asian, and others. The vast majority of the study participants were correctional officers (70.5%), followed by support staff, which included administrative staff, maintenance staff, food service staff, teachers, chaplains (11.7%), counselors (5.7%), medical staff (4.6%), lieutenants (4.3%), and others (3.2%). In general, the present study’s participants were not considerably different from the correction workers from previous studies [40,41] in terms of their demographic characteristics, such as mean age, proportion of male workers, and average job tenure.

The total number of correction workers at the two correctional facilities in the present study was 862, and the number of total respondents in the final data set was 353 after removing the cases of incomplete responses (i.e., more than 50% missing responses to survey; no response to the key study variables), suggesting that the minimum response rate was 41.0%. This response rate might be partly explained by the fact that correction workers often engage in shift work, are already dedicating a high degree of their attention to the volatile and dangerous environments they work in, and experience high level of fatigue from this demanding work, all of which contribute to their lack of availability and interest in participating in scientific studies. It can be noted that extant research on correction worker samples reports similar response rates of 37% [40] and 43% [42].

### 2.2. Measures

Psychosocial and behavioral variables judged to be potential responses to stressful and demanding working conditions were selected for Bayesian Network modeling. Physical assessment variables such as systolic/diastolic blood pressure, waist circumference, hand grip strength, and body mass index were excluded because the primary focus of the present study was on psychosocial and behavioral factors of correction workers. Also, bio-physiological factors were excluded as they were more relevant to chronic disease, having indirect long-term negative effects across the lifespan.

#### 2.2.1. Exhaustion

Work-related fatigue and exhaustion were assessed with three items adopted from the Burnout scale [43] and a 1–7 response scale (1: strongly disagree, 7: strongly agree). A sample item was; “After work, I have enough energy for leisure activities (reverse scored)”. Internal consistency was satisfactory with α = 0.70 [44].

#### 2.2.2. Disengagement

Correction workers’ perception of disengagement from their work was assessed with the two items adopted from the Burnout scale [43] and a 1–7 response scale (1: strongly disagree, 7: strongly agree). The items were; “More and more often I talk about my work in a negative way” and “Sometimes I feel really disgusted with my work.” Internal consistency was satisfactory (α = 0.82).

#### 2.2.3. Depression

Depressed mood was measured with the 10 items of the Center for Epidemiologic Studies Depression Scale [45]. All items had a 1–4 response scale (1: rarely or none of the time, less than 1 day per week, 4: all of the time, 5–7 days per week). An example item was; “I was bothered by things that usually don’t bother me.” Internal consistency was satisfactory (α = 0.76).

#### 2.2.4. Stress

A slightly adapted single-item version of the Stress in General Scale, which was shown to be valid by Stanton et al. [46], was utilized to assess stress. The item “On average, how stressful is your work?” had a 1–5 response scale (1: not at all, 5: extremely).

#### 2.2.5. Limitations to Regular Physical Leisure Exercise

Based on a series of focus group meetings with correction workers, and brainstorming with subject matter experts in psychophysical health, such as medical doctors, epidemiologists, ergonomists, as well as psychologists, a list of specific psychosocial and behavioral factors that are associated with workers’ motivation and engagement to physical exercises was created by the CPH-NEW research team [47]. Using a yes-no answer format, 12 factors that limit correction workers’ ability to get regular physical exercise were evaluated. Example items included “Fatigue or need to schedule sleep,” “Pain in joints or muscles,” and “Overtime or shift work.”

#### 2.2.6. Work–Family Balance (Work to Family and Family to Work Conflict)

Perceived level of conflict between demands from work and family was assessed with four items based on a 1–4 response scale (1: never, 4: always). The items were adopted from the questions used by Frone et al. [48]. An example item was “How often do things going on at work make you feel tense and irritable at home?” Internal consistency was satisfactory (α = 0.75).

#### 2.2.7. Workability

The Work Ability Index [49] was utilized to assess the correction workers’ perceived ability to perform their work. The index consists of four items that are calibrated on a 0–10 response scale (0: cannot work, 10: work at best). An example item was “How many points would you give your current ability to work?”. Internal consistency was satisfactory (α = 0.91).

### 2.3. Analysis

Bayesian Network modeling was performed with GeNie [50], which is freely accessible to researchers and practitioners. The study variables were all discretized into five equal intervals by percent distribution (i.e., 0–20%, 21–40%, 41–60%, 61–80%, and 81–100%) in order to allow consistency in making interpretations and to simplify the probabilistic dependency relationship between all possible study variable pairs. By doing so, the strength of the probabilistic link between two variables X and Y is defined as the chance of the specific state of Y (e.g., top 20 percentile) given the specific state of X (e.g., bottom 20 percentile) in the identified Bayesian Network model. Furthermore, the presumption is that a randomly-selected response category will be correct 20% of the time by chance (i.e., one correct response out of five response options). Thus, if the classification accuracy of each study variable’s state based on the Bayesian Network model is substantially greater than 20% (and closer to 100%), it can be concluded that the model is valid.

In the Bayesian Network analysis framework, “learning” represents successful estimation of probabilistic parameters and structure. After first randomly selecting half of the data (*n* = 182, 51.6%), a Bayesian Network model was learned without any prior assumptions about the interrelations among the interested psychosocial and behavioral factors. This enabled the modeling process to be completely data driven. Specifically, the “learn parameter” function of GeNie [50] was utilized; it is based on an expectation maximization (EM) algorithm with random restarts. EM is an iterative “hill-climbing” algorithm, which attempts to maximize the expected log likelihood of the probabilistic model [51,52]. It is one of the most widely used Bayesian Network learning techniques, and is particularly useful when data is incomplete (i.e., missing values) or when hidden variables are assumed.

The identified Bayesian Network model (learned with the first half of the data) was validated by testing the variables’ state classification accuracy using the second half of the data (*n* = 171, 48.4%). Specifically, leave-one-out (LOO) cross-validation was utilized because it does not waste any data points in contrast to other cross-validation approaches (e.g., k-fold cross-validation). The LOO cross-validation attempts to answer how accurately the learned Bayesian Network model predicts the state of a left-out data point given the second data set minus one data point. Accuracy is computed as the percent of correct predictions, which can be compared with the minimum accuracy level, referred to as the random classification accuracy [53].

### 2.4. Ethical Approval

All subjects gave their informed consent for inclusion before they participated in the study. The study was conducted in accordance with the Declaration of Helsinki, and the protocol was approved by the Internal Review Board (IRB) of UConn Health (IE-13-033S-2).

## 3. Results

### 3.1. Bayesian Network Model Specification

Descriptive statistics of the study variables as well as their inter-correlations are presented in Table 1. Bayesian Network model was “learned” to reveal the mutual dependence of the seven psychosocial and behavioral factors. The final Bayesian Network model is presented in Figure 1. This model represents the most probable scenario of how these factors are jointly associated with the health and well-being of correction workers.

### 3.2. Validation of the Bayesian Network Model

In the final Bayesian Network model, the accuracy of the study variables’ state classification was well above the random classification level (in the present study, 20%), suggesting acceptable, but somewhat less than optimal, model validity. The specific classification accuracies were: 31.0% (3.7–65.9% across the states) for exhaustion, 35.7% (12.5–63.2% across the states) for disengagement, 27.5% (0–46.7% across the states) for stress, 41.5% (4.5–74.5% across the states) for depression, 56.1% (0–72.4% across the states) for work-family balance, 38.6% (0–66.7% across the states) for limitations to regular physical leisure exercise, and 40.9% (0–66.1% across the states) for workability. These accuracy levels were all greater than the random state, even though variations were detected across different states of each study variable. Relatively low accuracy levels for particular states of the study variables were primarily due to small observations for these particular states of the study variables, either in the learning or validation data sets.

### 3.3. Conditional Probabilities

The final Bayesian Network model suggested that work-related exhaustion may lead to derivative occupational stress as well as a loss of workability, and that work-related exhaustion also limits a correction worker’s ability to get regular physical exercise. These three outcomes (i.e., stress, workability, and limitations to regular physical leisure exercise) were also consistently interrelated with the psychosocial factors of depressed mood, disengagement, and lack of work-family balance. Particularly, depressed mood served as an important “hub factor”, being directly related to most of the study variables. The likelihood of having a high (top 20 percentile) depressed mood increased from 6 to 38% as the level of work-related exhaustion changed from low (bottom 20 percentile) to high (top 20 percentile). The likelihood of having poor (bottom 20 percentile) workability increased from 4% to 48%, as the level of depressed mood increased from low (bottom 20 percentile) to high (top 20 percentile). The likelihood of having poor (bottom 20 percentile) ability to get regular physical exercise increased from 4 to 17%. Graphical representation of the study variables’ conditional probability distribution based on the final Bayesian Network model is presented in Figure 2.

The establishment of a final model permits the simulation of many different “what if” scenarios, allowing the researcher to explore any revealed relationship in greater detail. In the present study, the probabilities of correction workers’ stress, workability, and limitations to regular physical leisure exercise were calculated for two specific situations: (1) when other variables, such as exhaustion, disengagement, depression, and work–family balance are at their poorest (worst case scenario), and (2) when these same variables are optimal (best case scenario). As expected, the more desirable states of these variables—low stress, high workability, and reduced limitations to regular physical leisure exercise—were much more probable in the best case scenario than in the worst case scenario (Table 2).

## 4. Discussion

Psychological and physical health problems and concerns are diverse and prevalent among correction workers. In order to reveal the interplay among potential outcomes within their unique occupational context in a manner consistent with socio-technical systems approaches, the present study adopted the Bayesian Network analytic framework because of its flexibility and efficiency in exploring all possible interrelations among the study variables. Guided by well-established frameworks for workplace stress mechanisms, including the Job Demands-Resource Model [12], Spillover Theory [22,32], and Conservation of Resources Theory [23], study variables were selected based on assumptions about their potential interrelations. Subsequently, use of the data-driven Bayesian Network approach identified a model showing a probable scenario in which correction workers’ exhaustion had dominant influence over a chain reaction that significantly raised the risk of several other negative psychosocial and behavioral outcomes. Correction workers’ exhaustion was found to be associated with a lack of work engagement, depressed mood, and also interfered with work-family balance. Additionally, increased disengagement was found to be associated with work-related stress, while depressed mood was closely associated with less regular physical activity. At the same time, depressed mood and work-family imbalance were jointly associated with reduced work ability.

The results extend the previous finding of a close relationship between psychological distress and work-family imbalance among correction workers [54]. The identified joint processes of how the impact of exhaustion can be enhanced or mitigated by other factors also extends to previous findings regarding the impact of exhaustion on depression, absenteeism, depersonalization, and reduced personal accomplishment that were based on a French correctional officer sample [55].

### 4.1. Theoretical and Analytic Implications

First, the study findings complement relationships predicted by the Job Demands-Resource Model. In this model, job demands that result in exhaustion can negatively impact worker engagement, and this in turn is associated with work-related stress. Also, this model suggests that job demands that are closely associated with exhaustion will be negatively associated with psychosocial (i.e., mood and work-family imbalance) and organizational behavior outcomes (i.e., work ability). These same relationships were supported by the data-driven Bayesian Network model identified in the present study.

However contrary to relationships posited in the conventional stress models, relationships between stress to outcomes (i.e., engagement, workability) were not detected in the present study. This may be partly explained by the fact that other factors, such as depressed mood and work-family imbalance, were considered simultaneously along with the engagement and stress variables, speaking to the importance of examining psychosocial variables in combination with occupational context variables. Our findings indicate that the Job Demand-Resource Model may be a reasonable framework to first approach occupational health and safety issues, but it is also important to account for workers’ adjustment to their particular work context, as is the case in the present study of correction workers.

Second, although a stress-to-burnout directional relationship has been widely accepted in previous studies [8,9], the present study showed that burnout (i.e., exhaustion) may be associated with derivative stress, which is subsequent to employee disengagement. In fact, there have been previous reports that disengagement, which may be followed by burnout, leads to more stress in workplace [56] and also a distressed mental state [57]. It should be noted that the finding here of a burnout-to-stress relationship doesn’t necessarily reject the well-established causal link from stress to burnout, and also that our findings were based on the unique context of correctional work for which well-established causal linkages may not apply. Nonetheless, the results do suggest the need for adopting an unbiased perspective on how stress may be exacerbated by burnout in a particular population of workers. Within an autoregressive model framework [58], an outcome at one point in time can serve as a cause at another future point in time, and this may also be the case for the reciprocal stress and burnout relationship. Future studies could take a longitudinal approach to further examine the nature of the causal mechanisms behind the stress-burnout relationship.

### 4.2. Practical Implications

The results support use of the Total Worker Health framework for considering a wide range of factors impacting worker health and wellbeing. Integrated interventions that promote safety, health, and well-being among correction workers appear to be warranted, given the complex interplay of the psychosocial and behavioral factors reported on here. The results also suggest that taking steps to reduce the level of exhaustion in this working population may provide the most efficient way to reduce depressed mood, interruption of work-family balance, and a lack of work engagement. These factors were also shown to increase the risks of work-related stress, a reduction of regular physical exercise, and lowered work ability. Continued failure to manage workers’ exhaustion would be ignoring an apparent primary risk factor.

However, management of this primary risk factor may prove particularly challenging if there is short staffing and also overtime requirements in an organization that functions 24/7 and that cannot afford to be understaffed at any time. If work-related exhaustion cannot be avoided, then intervention efforts can next focus on the proper management of workers’ depressed mood because this was also shown to be strongly associated with the loss of work-family balance, increased negative attitudes toward their job, and reduced workability. Establishing a hierarchy of risk factors and then selecting the most attainable intervention is compatible with a participatory approach to intervention planning [28] which aims at identifying and addressing workers’ needs and concerns that are most salient given their present circumstances.

### 4.3. Limitations and Suggestions for Future Study

Some limitations of the present study need to be addressed in future research. Generalizability of the findings is limited given the uniqueness and relatively small size of the sample. In particular, it can be noted that only half of the data was utilized for Bayesian Network model learning, while the remaining half of the data was utilized for the validation of the learned Bayesian Network model. Also, the present study was cross-sectional, which did not allow examination of the dynamic relationships among study variables across time. Moreover, the final Bayesian Network model’s accuracy level was not at an ideal level, although it was found to be meaningfully above chance. To resolve this issue, a larger sample can be used for Bayesian Network model learning to enable more robust and reliable modeling. More data points generally ensure more reliable estimation of probability, particularly when the probability is smaller. Also, future studies are needed to more clearly demonstrate the distinct roles of job demands, job control, and support in the extended mechanisms affecting stressors, stress, strain, and the exacerbation of the strain symptoms. The role of individual differences, such as gender, age, and tenure in the interrelations among stress and stress outcomes can also be examined in future studies.

## 5. Conclusions

The present study demonstrated the value of using a machine learning algorithm, like Bayesian Network analysis, to explore the complicated interrelations among multiple psychosocial and behavioral factors in a specific socio-technical work context, and the ways these interrelations may be contributing to correctional officer health and wellness. Regardless of some limitations, like the relatively small sample size for a machine learning approach and the use of cross-sectional data, the findings of the present study suggest the importance of joint consideration of psychosocial and behavioral factors when investigating variables that may impact the health and wellbeing of correction workers. Moreover, the present study supported the value of adopting a Total Worker Health framework with integrated intervention approaches as a means to benefit workers in high-risk occupations.

## Figures and Tables

**Figure 1:**
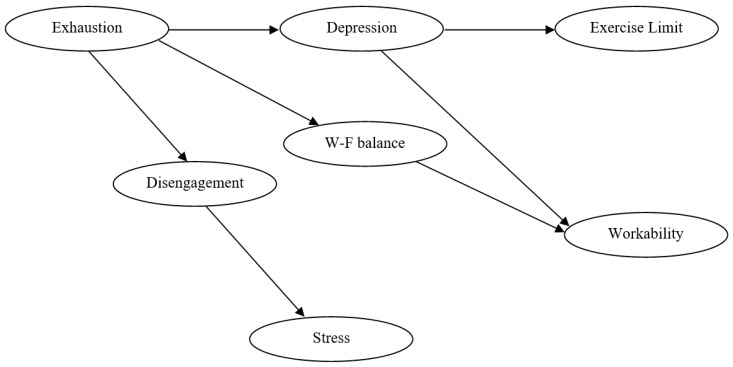
Final Bayesian Network model: the most probable scenario of the potential negative outcomes of correctional work. notes: W-F balance = Work-Family balance (work to family and family to work conflict); Exercise Limit = Limitations to regular physical leisure exercise.

**Figure 2:**
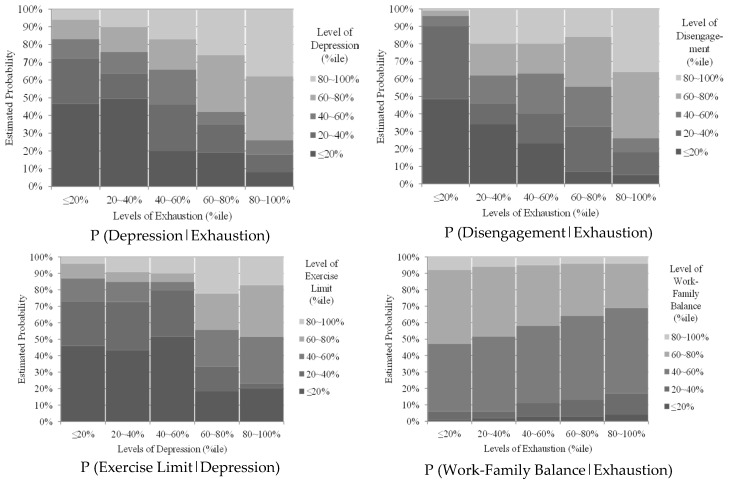
Conditional probability distribution based on the data-driven final Bayesian Network model. Notes: P(Y|X) = conditional probability of Y given X (i.e., probability of a particular state of Y given the particular state of X); Exercise Limit = Limitations to regular physical leisure exercise.

**Table 1:** Means, Standard deviations (SD), and correlations of the study variables.

Study Variables	Mean (SD)	1	2	3	4	5	6	7	8	9	10
1. Exhaustion	30.88 (10.21)	-									
2. Disengagement	30.82 (10.53)	0.49 **	-								
3. Depression	10.63 (0.44)	0.51 **	0.40 **	-							
4. Stress	10.56 (10.02)	0.47 **	0.46 **	0.34 **	-						
5. Exercise Limit	20.36 (10.73)	0.40 **	0.29 **	0.40 **	0.22 **	-					
6. Work-Family balance	30.37 (0.79)	−0.40 **	−0.24 **	−0.45 **	−0.27 **	−0.23 **	-				
7. Workability	80.76 (10.40)	−0.38 **	−0.30 **	−0.54 **	−0.32 **	−0.35 **	0.43 **	-			
8. Healthy diet	20.95 (0.54)	−0.18 **	−0.10 *^ns^*	−0.19 **	−0.05 *^ns^*	−0.31 **	0.15 **	0.09 *^ns^*	-		
9. Nutrition	20.58 (0.84)	−0.18 **	−0.14 *	−0.29 **	−0.02 *^ns^*	−0.28 **	0.21 **	0.16 **	0.51 **	-	
10. Readiness to Improve Health	30.80 (10.00)	−0.17 **	−0.14 **	−0.27 **	−0.04 *^ns^*	−0.24 **	0.28 **	0.26 **	0.44 **	0.49 **	-

Notes: Exercise limit = Limitations to regular physical leisure exercise; ** *p* < 0.01; * *p* < 0.05; *^ns^ p* < 0.01.

**Table 2:** Probabilities at the Simulated Worst and Best Scenarios.

Outcome Variables	At Worst Scenario	At Best Scenario
Exercise Limit	Low (Bottom 20 percentile) = 20%	Low (Bottom 20 percentile) = 49%
Workability	High (Top 40 percentile) = 1%	High (Top 40 percentile) = 89%
Stress	Low (Bottom 20 percentile) = 3%	Low (Bottom 20 percentile) = 52%

Notes: Low = Bottom 20 percentile; High = Top 20 percentile; Exercise Limit = Limitations to regular physical leisure exercise.
